# *N*′-[(*E*)-4-Hy­droxy­benzyl­idene]-2-(naph­tha­len-2-yl­oxy)acetohydrazide

**DOI:** 10.1107/S1600536812038408

**Published:** 2012-09-12

**Authors:** Rajni Kant, Vivek K. Gupta, Kamini Kapoor, S. Samshuddin, B. Narayana, B. K. Sarojini

**Affiliations:** a X-ray Crystallography Laboratory, Post-Graduate Department of Physics & Electronics, University of Jammu, Jammu Tawi 180 006, India; b Department of Studies in Chemistry, Mangalore University, Mangalagangotri 574 199, India; c Department of Chemistry, P.A. College of Engineering, Nadupadavu, Mangalore 574 153, India

## Abstract

The asymmetric unit of the title compound, C_19_H_16_N_2_O_3_, contains two independent mol­ecules in which the dihedral angles between the naphthalene ring system and the benzene ring are 10.0 (1) and 35.3 (1)°. In the crystal, mol­ecules are linked by N—H⋯O and O—H⋯O hydrogen bonds, forming a two-dimensional framework parallel to (001). Weak C—H⋯O and C—H⋯N hydrogen bonds complete a three-dimensional network.

## Related literature

For the pharmacological importance of Schiff base hydrazones, see: Rollas & Kucukguzel (2007 ▶). For related structures of Schiff base hydrazones, see: Fun *et al.* (2012*a* ▶,*b* ▶); Dutkiewicz *et al.* (2011 ▶); Narayana *et al.* (2007 ▶), Sarojini *et al.* (2007*a* ▶,*b* ▶,*c* ▶); Yathirajan *et al.* (2007*a* ▶,*b* ▶); Huang (2009 ▶).
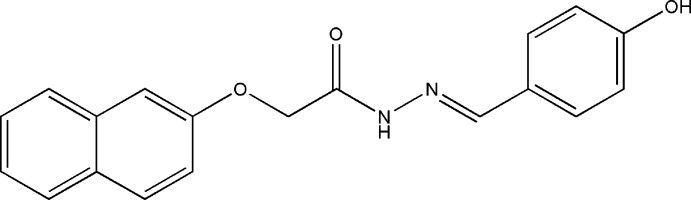


## Experimental

### 

#### Crystal data

C_19_H_16_N_2_O_3_
*M* *_r_* = 320.34
Orthorhombic, 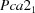

*a* = 17.2908 (8) Å
*b* = 6.9946 (3) Å
*c* = 27.1617 (11) Å
*V* = 3285.0 (2) Å^3^
*Z* = 8
Mo *K*α radiation
μ = 0.09 mm^−1^
*T* = 293 K
0.3 × 0.2 × 0.2 mm

#### Data collection

Oxford Diffraction Xcalibur Sapphire3 diffractometer
Absorption correction: multi-scan (*CrysAlis PRO*; Oxford Diffraction, 2010 ▶) *T*_min_ = 0.899, *T*_max_ = 1.000
19652 measured reflections
3632 independent reflections
2583 reflections with *I* > 2σ(*I*)
*R*_int_ = 0.042

#### Refinement

*R*[*F* ^2^ > 2σ(*F* ^2^)] = 0.049
*wR*(*F* ^2^) = 0.112
*S* = 1.07
3632 reflections
449 parameters
5 restraints
H atoms treated by a mixture of independent and constrained refinement
Δρ_max_ = 0.15 e Å^−3^
Δρ_min_ = −0.14 e Å^−3^

### 

Data collection: *CrysAlis PRO* (Oxford Diffraction, 2010 ▶); cell refinement: *CrysAlis PRO*; data reduction: *CrysAlis PRO* (Oxford Diffraction, 2010 ▶); program(s) used to solve structure: *SHELXS97* (Sheldrick, 2008 ▶); program(s) used to refine structure: *SHELXL97* (Sheldrick, 2008 ▶); molecular graphics: *ORTEP-3* (Farrugia, 1997 ▶); software used to prepare material for publication: *PLATON* (Spek, 2009 ▶).

## Supplementary Material

Crystal structure: contains datablock(s) I, New_Global_Publ_Block. DOI: 10.1107/S1600536812038408/lh5528sup1.cif

Structure factors: contains datablock(s) I. DOI: 10.1107/S1600536812038408/lh5528Isup2.hkl

Supplementary material file. DOI: 10.1107/S1600536812038408/lh5528Isup3.cml

Additional supplementary materials:  crystallographic information; 3D view; checkCIF report

## Figures and Tables

**Table 1:** Hydrogen-bond geometry (Å, °)

*D*—H⋯*A*	*D*—H	H⋯*A*	*D*⋯*A*	*D*—H⋯*A*
O7*A*—H7*A*⋯O11*B* ^i^	0.82 (3)	1.89 (4)	2.613 (5)	145 (5)
O7*B*—H7*B*⋯O11*A* ^ii^	0.83 (4)	1.82 (4)	2.642 (4)	170 (4)
N10*B*—H10*B*⋯O7*A* ^iii^	0.87 (2)	2.25 (2)	3.036 (5)	151 (1)
C3*B*—H3*B*⋯N9*A* ^ii^	0.93	2.46	3.368 (6)	166
C12*A*—H12*B*⋯O11*B* ^iv^	0.97	2.58	3.463 (5)	151
C22*A*—H22*A*⋯O11*B* ^iv^	0.93	2.60	3.391 (5)	144
C22*B*—H22*B*⋯O11*A* ^v^	0.93	2.56	3.390 (5)	149

Symmetry codes: (i) 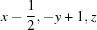; (ii) 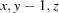; (iii) 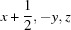; (iv) 
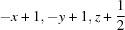
; (v) 
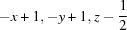
.

**Fig. 1.**
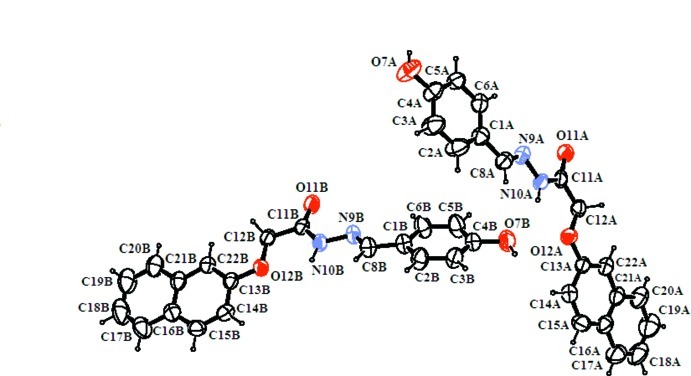
ORTEP-3 view (Farrugia, 1997) of the asymmetric unit. The ellipsoids are drawn at the 40% probability level. H atoms are shown as small spheres of arbitrary radii.

**Fig. 2.**
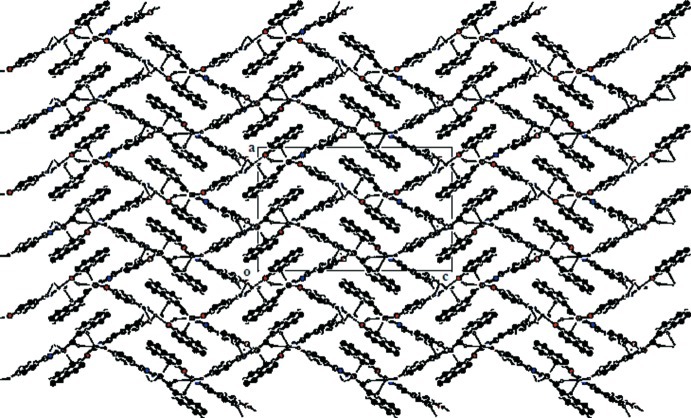
A molecular packing view of the title compound along the b axis, showing hydrogen bonds (dashed lines). For clarity, hydrogen atoms which are not involved in hydrogen bonding have been omitted.

## supplementary crystallographic information

### Comment

The pharmacological importance of Schiff base hydrazones are well documented (Rollas & Kucukguzel, 2007). The crystal structure of some Schiff base hydrazones *viz. N*-[(*E*)-4-chlorobenzylidene]pyridine-4-carbohydrazide monohydrate (Fun *et al.*,2012*a*),*N*'-(2,6-difluorobenzylidene) pyridine-4-carbohydrazide (Fun *et al.*,2012*b*),two new Schiff base hydrazones derived from biphenyl-4-carbohydrazide (Dutkiewicz *et al.*, 2011),2-bromo-*N*'-[(*E*)-(4-fluorophenyl)methylene]-5-methoxybenzohydrazide monohydrate (Narayana *et al.*,2007),2-bromo-*N*'-[(*E*)-4-hydroxybenzylidene] -5-methoxybenzohydrazide (Sarojini *et al.*,2007*a*),*N*'-isopropylidene- 6-methoxy-2-naphthohydrazide (Sarojini *et al.*,2007*b*), 2-bromo-N'-iso propylidene-5-methoxybenzohydrazide (Sarojini *et al.*,2007*c*), 2-bromo-5-methoxy-*N*'-[(*E*)-(2-nitrophenyl)methylene]benzohydrazide (Yathirajan *et al.*, 2007*a*)and *N*'-[(1E)-(4-fluorophenyl)methylene] -6-methoxy-2-naphthohydrazide (Yathirajan *et al.*, 2007*b*) have been reported. In view of the importance of Schiff base hydrazones, the title compound (I) is prepared and its crystal structure is reported.

The asymmetric unit of the title compound comprises of two crystallographically independent molecules, A and B (Fig. 1). The geometry of both independent molecules indicates a high degree of similarity in terms of their bond distances and bond angles and are comparable with a similar crystal structure (Huang, 2009). The dihedral angle between naphthalene ring system and benzene benzene ring is 10.0 (1)° in molecule A and 35.3 (1)° in molecule B. In the crystal, molecules are connected *via* O—H···O, N—H···O, weak C—H···O and weak C—H···N hydrogen bonds into a three-dimensional supramolecular structure.

### Experimental

A mixture of 2-(naphthalen-2-yloxy)acetohydrazide (0.216 g, 0.001 mol) and 4-hydroxybenzaldehyde (0.122 g, 0.001 mol) in 30 ml ethanol containing 2 drops of concentrated sulfuric acid was refluxed for about 3 h. On cooling solid was separated which was filtered and recrystallized from ethanol. The yield was 82%. (m.p. 436 K). The single-crystal was grown from a solution of the title compound in DMF by the slow evaporation method.

### Refinement

The N and O-bound H atoms were located in a difference Fourier map and refined independently with the constraints N—H = 0.86 (1) and O—H = 0.82 (1) Å. All other H atoms were positioned geometrically and were treated as riding on their parent C atoms, with C—H distances of 0.93–0.97 Å and with *U*_iso_(H) = 1.2*U*_eq_(C). In the absence of significant anomalous dispersion effects the Friedel pairs were merged.

### Crystal data

**Table:**

C_19_H_16_N_2_O_3_	*F*(000) = 1344
*M**_r_* = 320.34	*D*_x_ = 1.295 Mg m^−^^3^
Orthorhombic, *P**c**a*2_1_	Mo *K*α radiation, λ = 0.71073 Å
Hall symbol: P 2c -2ac	Cell parameters from 6059 reflections
*a* = 17.2908 (8) Å	θ = 3.5–29.0°
*b* = 6.9946 (3) Å	µ = 0.09 mm^−^^1^
*c* = 27.1617 (11) Å	*T* = 293 K
*V* = 3285.0 (2) Å^3^	Block, white
*Z* = 8	0.3 × 0.2 × 0.2 mm

### Data collection

**Table:**

Oxford Diffraction Xcalibur Sapphire3 diffractometer	3632 independent reflections
Radiation source: fine-focus sealed tube	2583 reflections with *I* > 2σ(*I*)
Graphite monochromator	*R*_int_ = 0.042
Detector resolution: 16.1049 pixels mm^-1^	θ_max_ = 27.0°, θ_min_ = 3.5°
ω scans	*h* = −21→21
Absorption correction: multi-scan (*CrysAlis PRO*; Oxford Diffraction, 2010)	*k* = −8→8
*T*_min_ = 0.899, *T*_max_ = 1.000	*l* = −34→30
19652 measured reflections	

### Refinement

**Table:**

Refinement on *F*^2^	Primary atom site location: structure-invariant direct methods
Least-squares matrix: full	Secondary atom site location: difference Fourier map
*R*[*F*^2^ > 2σ(*F*^2^)] = 0.049	Hydrogen site location: inferred from neighbouring sites
*wR*(*F*^2^) = 0.112	H atoms treated by a mixture of independent and constrained refinement
*S* = 1.07	*w* = 1/[σ^2^(*F*_o_^2^) + (0.0343*P*)^2^ + 1.0102*P*] where *P* = (*F*_o_^2^ + 2*F*_c_^2^)/3
3632 reflections	(Δ/σ)_max_ = 0.001
449 parameters	Δρ_max_ = 0.15 e Å^−^^3^
5 restraints	Δρ_min_ = −0.14 e Å^−^^3^

### Special details

**Table:**

Experimental. *CrysAlis PRO*, Oxford Diffraction Ltd., Version 1.171.34.40 (release 27–08-2010 CrysAlis171. NET) (compiled Aug 27 2010,11:50:40) Empirical absorption correction using spherical harmonics, implemented in SCALE3 ABSPACK scaling algorithm.
Geometry. All e.s.d.'s (except the e.s.d. in the dihedral angle between two l.s. planes) are estimated using the full covariance matrix. The cell e.s.d.'s are taken into account individually in the estimation of e.s.d.'s in distances, angles and torsion angles; correlations between e.s.d.'s in cell parameters are only used when they are defined by crystal symmetry. An approximate (isotropic) treatment of cell e.s.d.'s is used for estimating e.s.d.'s involving l.s. planes.
Refinement. Refinement of *F*^2^ against ALL reflections. The weighted *R*-factor *wR* and goodness of fit *S* are based on *F*^2^, conventional *R*-factors *R* are based on *F*, with *F* set to zero for negative *F*^2^. The threshold expression of *F*^2^ > σ(*F*^2^) is used only for calculating *R*-factors(gt) *etc*. and is not relevant to the choice of reflections for refinement. *R*-factors based on *F*^2^ are statistically about twice as large as those based on *F*, and *R*- factors based on ALL data will be even larger.

### Fractional atomic coordinates and isotropic or equivalent isotropic displacement parameters (Å^2^)

**Table:**

	*x*	*y*	*z*	*U*_iso_*/*U*_eq_	
C1A	0.2482 (2)	0.4059 (6)	−0.14893 (15)	0.0502 (10)	
C2A	0.2422 (3)	0.2457 (7)	−0.17829 (19)	0.0699 (13)	
H2A	0.2634	0.1307	−0.1676	0.084*	
C3A	0.2052 (3)	0.2536 (7)	−0.22309 (19)	0.0743 (14)	
H3A	0.2022	0.1451	−0.2428	0.089*	
C4A	0.1728 (2)	0.4225 (6)	−0.23867 (17)	0.0546 (11)	
C6A	0.2168 (2)	0.5745 (6)	−0.16513 (15)	0.0546 (10)	
H6A	0.2210	0.6841	−0.1459	0.066*	
C5A	0.1786 (2)	0.5827 (6)	−0.21025 (16)	0.0532 (10)	
H5A	0.1571	0.6972	−0.2210	0.064*	
O7A	0.1354 (2)	0.4179 (5)	−0.28308 (13)	0.0770 (10)	
C8A	0.2889 (2)	0.3847 (6)	−0.10197 (15)	0.0513 (10)	
H8A	0.3009	0.2622	−0.0911	0.062*	
N9A	0.3085 (2)	0.5267 (5)	−0.07535 (14)	0.0510 (9)	
N10A	0.3504 (2)	0.4753 (5)	−0.03367 (13)	0.0447 (8)	
C11A	0.3730 (2)	0.6093 (6)	−0.00186 (14)	0.0459 (9)	
O11A	0.35586 (19)	0.7796 (4)	−0.00537 (11)	0.0636 (8)	
O12A	0.44014 (15)	0.3498 (4)	0.03806 (10)	0.0495 (7)	
C12A	0.4206 (2)	0.5452 (5)	0.04084 (16)	0.0480 (9)	
H12A	0.4677	0.6205	0.0421	0.058*	
H12B	0.3922	0.5682	0.0710	0.058*	
C13A	0.4888 (2)	0.2836 (6)	0.07439 (14)	0.0442 (9)	
C14A	0.5162 (2)	0.0966 (6)	0.06702 (16)	0.0535 (10)	
H14A	0.5011	0.0271	0.0395	0.064*	
C15A	0.5655 (3)	0.0182 (6)	0.1010 (2)	0.0632 (14)	
H15A	0.5823	−0.1071	0.0969	0.076*	
C16A	0.5912 (2)	0.1234 (7)	0.14197 (16)	0.0582 (11)	
C17A	0.6445 (3)	0.0489 (9)	0.1769 (2)	0.0816 (17)	
H17A	0.6626	−0.0757	0.1738	0.098*	
C18A	0.6692 (3)	0.1605 (11)	0.2151 (2)	0.0922 (18)	
H18A	0.7043	0.1104	0.2376	0.111*	
C19A	0.6430 (3)	0.3479 (10)	0.2214 (2)	0.0865 (16)	
H19A	0.6609	0.4215	0.2475	0.104*	
C20A	0.5910 (3)	0.4217 (8)	0.18875 (17)	0.0684 (13)	
H20A	0.5732	0.5462	0.1929	0.082*	
C21A	0.5638 (2)	0.3105 (6)	0.14854 (14)	0.0518 (10)	
C22A	0.5118 (2)	0.3884 (6)	0.11403 (14)	0.0496 (9)	
H22A	0.4931	0.5119	0.1183	0.059*	
C1B	0.4922 (2)	−0.0263 (6)	−0.19756 (15)	0.0502 (11)	
C2B	0.4561 (3)	−0.1702 (6)	−0.17164 (15)	0.0601 (11)	
H2B	0.4522	−0.2908	−0.1858	0.072*	
C3B	0.4257 (3)	−0.1410 (6)	−0.12522 (16)	0.0562 (11)	
H3B	0.4010	−0.2401	−0.1087	0.067*	
C4B	0.4324 (2)	0.0357 (6)	−0.10368 (17)	0.0523 (11)	
C5B	0.4652 (3)	0.1827 (6)	−0.12975 (18)	0.0682 (13)	
H5B	0.4676	0.3041	−0.1159	0.082*	
C6B	0.4949 (3)	0.1526 (6)	−0.17646 (18)	0.0640 (12)	
H6B	0.5167	0.2539	−0.1937	0.077*	
O7B	0.4058 (2)	0.0738 (5)	−0.05744 (12)	0.0690 (9)	
C8B	0.5265 (2)	−0.0703 (6)	−0.24509 (15)	0.0530 (10)	
H8B	0.5228	−0.1939	−0.2575	0.064*	
N9B	0.56127 (19)	0.0566 (5)	−0.26994 (13)	0.0487 (8)	
N10B	0.5977 (2)	−0.0100 (5)	−0.31289 (13)	0.0468 (9)	
C11B	0.6254 (2)	0.1164 (6)	−0.34475 (14)	0.0440 (9)	
C12B	0.6703 (2)	0.0412 (5)	−0.38801 (15)	0.0458 (9)	
H12C	0.6403	0.0597	−0.4178	0.055*	
H12D	0.7179	0.1134	−0.3913	0.055*	
C13B	0.7364 (2)	−0.2279 (5)	−0.41863 (14)	0.0428 (9)	
O11B	0.61664 (19)	0.2887 (4)	−0.34142 (11)	0.0640 (8)	
O12B	0.68813 (15)	−0.1539 (3)	−0.38294 (9)	0.0482 (6)	
C14B	0.7616 (2)	−0.4145 (6)	−0.41022 (15)	0.0513 (10)	
H14B	0.7457	−0.4797	−0.3822	0.062*	
C15B	0.8093 (3)	−0.5007 (6)	−0.44298 (19)	0.0552 (12)	
H15B	0.8250	−0.6261	−0.4374	0.066*	
C16B	0.8360 (2)	−0.4036 (6)	−0.48580 (16)	0.0530 (10)	
C17B	0.8876 (3)	−0.4868 (9)	−0.5205 (2)	0.0770 (17)	
H17B	0.9044	−0.6120	−0.5163	0.092*	
C18B	0.9122 (3)	−0.3839 (10)	−0.5597 (2)	0.0901 (18)	
H18B	0.9465	−0.4389	−0.5819	0.108*	
C19B	0.8870 (3)	−0.1978 (9)	−0.5674 (2)	0.0816 (16)	
H19B	0.9039	−0.1302	−0.5948	0.098*	
C20B	0.8381 (3)	−0.1149 (7)	−0.53511 (17)	0.0649 (12)	
H20B	0.8219	0.0102	−0.5405	0.078*	
C21B	0.8109 (2)	−0.2146 (6)	−0.49328 (14)	0.0492 (9)	
C22B	0.7597 (2)	−0.1282 (5)	−0.45945 (15)	0.0451 (9)	
H22B	0.7419	−0.0045	−0.4648	0.054*	
H10A	0.3612 (19)	0.3556 (19)	−0.0309 (14)	0.035 (10)*	
H10B	0.609 (2)	−0.131 (2)	−0.3160 (16)	0.052 (12)*	
H7B	0.394 (3)	−0.026 (5)	−0.043 (2)	0.10 (2)*	
H7A	0.126 (3)	0.530 (3)	−0.290 (2)	0.12 (3)*	

### Atomic displacement parameters (Å^2^)

**Table:**

	*U*^11^	*U*^22^	*U*^33^	*U*^12^	*U*^13^	*U*^23^
C1A	0.046 (2)	0.057 (3)	0.047 (3)	−0.0035 (19)	−0.0026 (18)	0.002 (2)
C2A	0.082 (3)	0.056 (3)	0.071 (3)	0.006 (2)	−0.033 (3)	−0.001 (2)
C3A	0.100 (4)	0.055 (3)	0.068 (3)	0.005 (2)	−0.033 (3)	−0.006 (2)
C4A	0.062 (3)	0.054 (2)	0.048 (3)	−0.010 (2)	−0.014 (2)	0.004 (2)
C6A	0.063 (3)	0.057 (2)	0.043 (3)	0.003 (2)	0.002 (2)	−0.003 (2)
C5A	0.056 (2)	0.054 (2)	0.050 (3)	0.0053 (19)	−0.0005 (19)	0.011 (2)
O7A	0.109 (3)	0.060 (2)	0.061 (2)	−0.0103 (19)	−0.036 (2)	0.0106 (18)
C8A	0.053 (2)	0.057 (2)	0.044 (2)	−0.0005 (19)	−0.0018 (18)	0.006 (2)
N9A	0.057 (2)	0.058 (2)	0.038 (2)	−0.0010 (16)	0.0012 (16)	0.0027 (17)
N10A	0.054 (2)	0.045 (2)	0.035 (2)	0.0009 (15)	−0.0021 (16)	0.0023 (16)
C11A	0.057 (2)	0.049 (2)	0.032 (2)	0.0011 (18)	0.0111 (17)	0.0004 (19)
O11A	0.101 (2)	0.0446 (16)	0.0455 (17)	0.0172 (15)	0.0071 (16)	−0.0022 (14)
O12A	0.0599 (16)	0.0472 (15)	0.0413 (15)	0.0050 (12)	−0.0073 (13)	−0.0036 (13)
C12A	0.058 (2)	0.050 (2)	0.036 (2)	−0.0013 (18)	0.0018 (19)	−0.0048 (19)
C13A	0.043 (2)	0.055 (2)	0.035 (2)	0.0013 (17)	0.0010 (16)	0.0016 (18)
C14A	0.056 (2)	0.057 (3)	0.047 (3)	0.002 (2)	0.006 (2)	−0.002 (2)
C15A	0.058 (3)	0.061 (3)	0.071 (4)	0.015 (2)	0.014 (3)	0.012 (3)
C16A	0.042 (2)	0.085 (3)	0.047 (3)	0.004 (2)	0.0051 (19)	0.014 (2)
C17A	0.057 (3)	0.112 (4)	0.076 (4)	0.018 (3)	0.005 (3)	0.028 (3)
C18A	0.060 (3)	0.158 (6)	0.059 (4)	0.009 (4)	−0.016 (3)	0.025 (4)
C19A	0.061 (3)	0.130 (5)	0.069 (3)	−0.011 (3)	−0.013 (3)	0.005 (4)
C20A	0.061 (3)	0.096 (4)	0.049 (3)	−0.012 (2)	−0.007 (2)	0.007 (3)
C21A	0.043 (2)	0.073 (3)	0.039 (2)	−0.0045 (19)	0.0028 (17)	0.005 (2)
C22A	0.050 (2)	0.058 (2)	0.040 (2)	−0.0047 (17)	0.0019 (18)	0.0021 (19)
C1B	0.048 (2)	0.056 (3)	0.046 (3)	−0.0055 (18)	0.0061 (18)	−0.008 (2)
C2B	0.080 (3)	0.053 (3)	0.047 (3)	−0.014 (2)	0.001 (2)	−0.012 (2)
C3B	0.074 (3)	0.052 (2)	0.042 (2)	−0.008 (2)	0.004 (2)	0.000 (2)
C4B	0.056 (2)	0.058 (3)	0.043 (2)	0.0069 (19)	0.008 (2)	0.000 (2)
C5B	0.087 (3)	0.055 (3)	0.063 (3)	−0.007 (2)	0.023 (3)	−0.016 (2)
C6B	0.075 (3)	0.052 (3)	0.065 (3)	−0.010 (2)	0.026 (2)	−0.007 (2)
O7B	0.098 (3)	0.057 (2)	0.052 (2)	0.0083 (18)	0.0201 (17)	−0.0010 (17)
C8B	0.057 (2)	0.057 (2)	0.045 (2)	−0.0069 (19)	0.0052 (19)	−0.010 (2)
N9B	0.0492 (19)	0.057 (2)	0.040 (2)	−0.0007 (16)	0.0070 (16)	−0.0064 (16)
N10B	0.055 (2)	0.047 (2)	0.038 (2)	0.0013 (15)	0.0037 (16)	−0.0056 (16)
C11B	0.053 (2)	0.046 (2)	0.033 (2)	0.0033 (17)	−0.0060 (17)	−0.0015 (18)
C12B	0.054 (2)	0.049 (2)	0.034 (2)	−0.0003 (17)	0.0010 (19)	0.0036 (18)
C13B	0.043 (2)	0.050 (2)	0.036 (2)	−0.0021 (17)	−0.0034 (15)	−0.0031 (18)
O11B	0.107 (2)	0.0440 (16)	0.0407 (17)	0.0083 (15)	0.0065 (16)	0.0015 (13)
O12B	0.0604 (17)	0.0442 (15)	0.0400 (15)	0.0033 (12)	0.0093 (12)	0.0018 (13)
C14B	0.054 (2)	0.055 (2)	0.045 (3)	0.0036 (19)	−0.0071 (19)	0.0054 (19)
C15B	0.055 (3)	0.053 (3)	0.057 (3)	0.0107 (19)	−0.013 (2)	−0.004 (2)
C16B	0.039 (2)	0.069 (3)	0.051 (3)	0.0026 (18)	−0.0093 (18)	−0.011 (2)
C17B	0.051 (3)	0.108 (4)	0.072 (4)	0.021 (3)	−0.004 (3)	−0.025 (3)
C18B	0.058 (3)	0.142 (5)	0.071 (4)	0.010 (3)	0.019 (3)	−0.028 (4)
C19B	0.064 (3)	0.117 (5)	0.064 (3)	−0.010 (3)	0.020 (3)	−0.005 (3)
C20B	0.061 (3)	0.080 (3)	0.054 (3)	−0.011 (2)	0.008 (2)	0.000 (2)
C21B	0.042 (2)	0.065 (3)	0.041 (2)	−0.0069 (18)	−0.0033 (17)	−0.0069 (19)
C22B	0.045 (2)	0.048 (2)	0.042 (2)	−0.0014 (17)	−0.0002 (17)	−0.0015 (19)

### Geometric parameters (Å, º)

**Table:**

C1A—C6A	1.371 (6)	C1B—C2B	1.377 (6)
C1A—C2A	1.380 (6)	C1B—C6B	1.377 (6)
C1A—C8A	1.464 (5)	C1B—C8B	1.454 (6)
C2A—C3A	1.375 (6)	C2B—C3B	1.382 (6)
C2A—H2A	0.9300	C2B—H2B	0.9300
C3A—C4A	1.375 (6)	C3B—C4B	1.372 (6)
C3A—H3A	0.9300	C3B—H3B	0.9300
C4A—C5A	1.364 (6)	C4B—O7B	1.364 (5)
C4A—O7A	1.369 (5)	C4B—C5B	1.372 (6)
C6A—C5A	1.393 (6)	C5B—C6B	1.385 (6)
C6A—H6A	0.9300	C5B—H5B	0.9300
C5A—H5A	0.9300	C6B—H6B	0.9300
O7A—H7A	0.825 (11)	O7B—H7B	0.826 (11)
C8A—N9A	1.275 (5)	C8B—N9B	1.267 (5)
C8A—H8A	0.9300	C8B—H8B	0.9300
N9A—N10A	1.391 (5)	N9B—N10B	1.405 (5)
N10A—C11A	1.333 (5)	N10B—C11B	1.327 (5)
N10A—H10A	0.861 (10)	N10B—H10B	0.868 (10)
C11A—O11A	1.231 (4)	C11B—O11B	1.218 (4)
C11A—C12A	1.492 (6)	C11B—C12B	1.504 (5)
O12A—C13A	1.377 (4)	C12B—O12B	1.405 (4)
O12A—C12A	1.410 (4)	C12B—H12C	0.9700
C12A—H12A	0.9700	C12B—H12D	0.9700
C12A—H12B	0.9700	C13B—C22B	1.370 (5)
C13A—C22A	1.362 (5)	C13B—O12B	1.380 (4)
C13A—C14A	1.405 (6)	C13B—C14B	1.395 (5)
C14A—C15A	1.371 (7)	C14B—C15B	1.355 (6)
C14A—H14A	0.9300	C14B—H14B	0.9300
C15A—C16A	1.406 (7)	C15B—C16B	1.424 (7)
C15A—H15A	0.9300	C15B—H15B	0.9300
C16A—C21A	1.403 (6)	C16B—C21B	1.406 (6)
C16A—C17A	1.423 (7)	C16B—C17B	1.422 (6)
C17A—C18A	1.366 (8)	C17B—C18B	1.353 (8)
C17A—H17A	0.9300	C17B—H17B	0.9300
C18A—C19A	1.397 (8)	C18B—C19B	1.388 (8)
C18A—H18A	0.9300	C18B—H18B	0.9300
C19A—C20A	1.364 (7)	C19B—C20B	1.349 (7)
C19A—H19A	0.9300	C19B—H19B	0.9300
C20A—C21A	1.421 (6)	C20B—C21B	1.413 (6)
C20A—H20A	0.9300	C20B—H20B	0.9300
C21A—C22A	1.409 (5)	C21B—C22B	1.412 (5)
C22A—H22A	0.9300	C22B—H22B	0.9300
			
C6A—C1A—C2A	118.9 (4)	C2B—C1B—C6B	117.8 (4)
C6A—C1A—C8A	123.9 (4)	C2B—C1B—C8B	118.9 (4)
C2A—C1A—C8A	117.2 (4)	C6B—C1B—C8B	123.2 (4)
C3A—C2A—C1A	120.9 (4)	C1B—C2B—C3B	122.1 (4)
C3A—C2A—H2A	119.5	C1B—C2B—H2B	119.0
C1A—C2A—H2A	119.5	C3B—C2B—H2B	119.0
C2A—C3A—C4A	119.8 (4)	C4B—C3B—C2B	119.4 (4)
C2A—C3A—H3A	120.1	C4B—C3B—H3B	120.3
C4A—C3A—H3A	120.1	C2B—C3B—H3B	120.3
C5A—C4A—O7A	123.6 (4)	O7B—C4B—C5B	117.9 (4)
C5A—C4A—C3A	120.1 (4)	O7B—C4B—C3B	122.7 (4)
O7A—C4A—C3A	116.3 (4)	C5B—C4B—C3B	119.3 (4)
C1A—C6A—C5A	120.3 (4)	C4B—C5B—C6B	120.8 (4)
C1A—C6A—H6A	119.8	C4B—C5B—H5B	119.6
C5A—C6A—H6A	119.8	C6B—C5B—H5B	119.6
C4A—C5A—C6A	119.9 (4)	C1B—C6B—C5B	120.5 (4)
C4A—C5A—H5A	120.0	C1B—C6B—H6B	119.8
C6A—C5A—H5A	120.0	C5B—C6B—H6B	119.8
C4A—O7A—H7A	107 (4)	C4B—O7B—H7B	110 (4)
N9A—C8A—C1A	122.9 (4)	N9B—C8B—C1B	121.2 (4)
N9A—C8A—H8A	118.6	N9B—C8B—H8B	119.4
C1A—C8A—H8A	118.6	C1B—C8B—H8B	119.4
C8A—N9A—N10A	113.5 (3)	C8B—N9B—N10B	115.0 (3)
C11A—N10A—N9A	119.9 (3)	C11B—N10B—N9B	118.8 (3)
C11A—N10A—H10A	124 (3)	C11B—N10B—H10B	120 (3)
N9A—N10A—H10A	116 (3)	N9B—N10B—H10B	120 (3)
O11A—C11A—N10A	124.0 (4)	O11B—C11B—N10B	124.5 (4)
O11A—C11A—C12A	118.9 (4)	O11B—C11B—C12B	117.9 (4)
N10A—C11A—C12A	117.0 (4)	N10B—C11B—C12B	117.6 (3)
C13A—O12A—C12A	115.7 (3)	O12B—C12B—C11B	112.1 (3)
O12A—C12A—C11A	112.4 (3)	O12B—C12B—H12C	109.2
O12A—C12A—H12A	109.1	C11B—C12B—H12C	109.2
C11A—C12A—H12A	109.1	O12B—C12B—H12D	109.2
O12A—C12A—H12B	109.1	C11B—C12B—H12D	109.2
C11A—C12A—H12B	109.1	H12C—C12B—H12D	107.9
H12A—C12A—H12B	107.8	C22B—C13B—O12B	123.7 (3)
C22A—C13A—O12A	124.3 (3)	C22B—C13B—C14B	121.1 (4)
C22A—C13A—C14A	121.0 (4)	O12B—C13B—C14B	115.2 (3)
O12A—C13A—C14A	114.6 (3)	C13B—O12B—C12B	115.3 (3)
C15A—C14A—C13A	119.1 (4)	C15B—C14B—C13B	120.0 (4)
C15A—C14A—H14A	120.5	C15B—C14B—H14B	120.0
C13A—C14A—H14A	120.5	C13B—C14B—H14B	120.0
C14A—C15A—C16A	121.3 (4)	C14B—C15B—C16B	121.4 (4)
C14A—C15A—H15A	119.3	C14B—C15B—H15B	119.3
C16A—C15A—H15A	119.3	C16B—C15B—H15B	119.3
C21A—C16A—C15A	118.8 (4)	C21B—C16B—C17B	118.9 (5)
C21A—C16A—C17A	118.4 (5)	C21B—C16B—C15B	117.8 (4)
C15A—C16A—C17A	122.8 (5)	C17B—C16B—C15B	123.3 (4)
C18A—C17A—C16A	120.0 (5)	C18B—C17B—C16B	120.0 (5)
C18A—C17A—H17A	120.0	C18B—C17B—H17B	120.0
C16A—C17A—H17A	120.0	C16B—C17B—H17B	120.0
C17A—C18A—C19A	121.8 (5)	C17B—C18B—C19B	121.2 (5)
C17A—C18A—H18A	119.1	C17B—C18B—H18B	119.4
C19A—C18A—H18A	119.1	C19B—C18B—H18B	119.4
C20A—C19A—C18A	119.3 (5)	C20B—C19B—C18B	120.1 (5)
C20A—C19A—H19A	120.3	C20B—C19B—H19B	119.9
C18A—C19A—H19A	120.3	C18B—C19B—H19B	119.9
C19A—C20A—C21A	120.7 (5)	C19B—C20B—C21B	121.2 (5)
C19A—C20A—H20A	119.7	C19B—C20B—H20B	119.4
C21A—C20A—H20A	119.7	C21B—C20B—H20B	119.4
C16A—C21A—C22A	119.4 (4)	C16B—C21B—C22B	120.1 (4)
C16A—C21A—C20A	119.8 (4)	C16B—C21B—C20B	118.5 (4)
C22A—C21A—C20A	120.7 (4)	C22B—C21B—C20B	121.4 (4)
C13A—C22A—C21A	120.3 (4)	C13B—C22B—C21B	119.6 (3)
C13A—C22A—H22A	119.9	C13B—C22B—H22B	120.2
C21A—C22A—H22A	119.9	C21B—C22B—H22B	120.2
			
C6A—C1A—C2A—C3A	−0.1 (8)	C6B—C1B—C2B—C3B	−2.3 (7)
C8A—C1A—C2A—C3A	−179.7 (5)	C8B—C1B—C2B—C3B	176.7 (4)
C1A—C2A—C3A—C4A	−1.0 (8)	C1B—C2B—C3B—C4B	−1.0 (7)
C2A—C3A—C4A—C5A	1.3 (8)	C2B—C3B—C4B—O7B	−178.0 (4)
C2A—C3A—C4A—O7A	−178.4 (5)	C2B—C3B—C4B—C5B	3.6 (7)
C2A—C1A—C6A—C5A	0.8 (6)	O7B—C4B—C5B—C6B	178.5 (4)
C8A—C1A—C6A—C5A	−179.7 (4)	C3B—C4B—C5B—C6B	−3.0 (7)
O7A—C4A—C5A—C6A	179.1 (4)	C2B—C1B—C6B—C5B	2.9 (7)
C3A—C4A—C5A—C6A	−0.6 (7)	C8B—C1B—C6B—C5B	−176.1 (4)
C1A—C6A—C5A—C4A	−0.4 (6)	C4B—C5B—C6B—C1B	−0.3 (7)
C6A—C1A—C8A—N9A	−10.6 (6)	C2B—C1B—C8B—N9B	−178.9 (4)
C2A—C1A—C8A—N9A	169.0 (4)	C6B—C1B—C8B—N9B	0.0 (7)
C1A—C8A—N9A—N10A	−176.5 (3)	C1B—C8B—N9B—N10B	174.3 (4)
C8A—N9A—N10A—C11A	−178.3 (4)	C8B—N9B—N10B—C11B	170.0 (4)
N9A—N10A—C11A—O11A	3.8 (6)	N9B—N10B—C11B—O11B	−5.7 (6)
N9A—N10A—C11A—C12A	−177.4 (3)	N9B—N10B—C11B—C12B	174.6 (3)
C13A—O12A—C12A—C11A	175.5 (3)	O11B—C11B—C12B—O12B	169.2 (3)
O11A—C11A—C12A—O12A	−175.8 (3)	N10B—C11B—C12B—O12B	−11.0 (5)
N10A—C11A—C12A—O12A	5.3 (5)	C22B—C13B—O12B—C12B	−6.8 (5)
C12A—O12A—C13A—C22A	6.7 (5)	C14B—C13B—O12B—C12B	172.4 (3)
C12A—O12A—C13A—C14A	−171.3 (3)	C11B—C12B—O12B—C13B	−173.3 (3)
C22A—C13A—C14A—C15A	1.4 (6)	C22B—C13B—C14B—C15B	−0.7 (6)
O12A—C13A—C14A—C15A	179.6 (4)	O12B—C13B—C14B—C15B	−179.9 (4)
C13A—C14A—C15A—C16A	−2.3 (6)	C13B—C14B—C15B—C16B	1.3 (6)
C14A—C15A—C16A—C21A	1.7 (6)	C14B—C15B—C16B—C21B	−0.3 (6)
C14A—C15A—C16A—C17A	−177.6 (4)	C14B—C15B—C16B—C17B	178.4 (4)
C21A—C16A—C17A—C18A	−1.4 (7)	C21B—C16B—C17B—C18B	0.5 (7)
C15A—C16A—C17A—C18A	177.9 (5)	C15B—C16B—C17B—C18B	−178.2 (5)
C16A—C17A—C18A—C19A	0.5 (8)	C16B—C17B—C18B—C19B	−1.1 (8)
C17A—C18A—C19A—C20A	0.5 (9)	C17B—C18B—C19B—C20B	1.1 (9)
C18A—C19A—C20A—C21A	−0.4 (8)	C18B—C19B—C20B—C21B	−0.5 (8)
C15A—C16A—C21A—C22A	−0.3 (6)	C17B—C16B—C21B—C22B	−180.0 (4)
C17A—C16A—C21A—C22A	179.1 (4)	C15B—C16B—C21B—C22B	−1.2 (5)
C15A—C16A—C21A—C20A	−177.9 (4)	C17B—C16B—C21B—C20B	0.0 (6)
C17A—C16A—C21A—C20A	1.5 (6)	C15B—C16B—C21B—C20B	178.8 (4)
C19A—C20A—C21A—C16A	−0.6 (6)	C19B—C20B—C21B—C16B	0.0 (6)
C19A—C20A—C21A—C22A	−178.2 (4)	C19B—C20B—C21B—C22B	180.0 (4)
O12A—C13A—C22A—C21A	−177.9 (3)	O12B—C13B—C22B—C21B	178.3 (3)
C14A—C13A—C22A—C21A	0.0 (6)	C14B—C13B—C22B—C21B	−0.9 (5)
C16A—C21A—C22A—C13A	−0.6 (6)	C16B—C21B—C22B—C13B	1.8 (5)
C20A—C21A—C22A—C13A	177.0 (4)	C20B—C21B—C22B—C13B	−178.2 (4)

### Hydrogen-bond geometry (Å, º)

**Table:**

*D*—H···*A*	*D*—H	H···*A*	*D*···*A*	*D*—H···*A*
O7*A*—H7*A*···O11*B*^i^	0.82 (3)	1.89 (4)	2.613 (5)	145 (5)
O7*B*—H7*B*···O11*A*^ii^	0.83 (4)	1.82 (4)	2.642 (4)	170 (4)
N10*B*—H10*B*···O7*A*^iii^	0.87 (2)	2.25 (2)	3.036 (5)	151 (1)
C3*B*—H3*B*···N9*A*^ii^	0.93	2.46	3.368 (6)	166
C12*A*—H12*B*···O11*B*^iv^	0.97	2.58	3.463 (5)	151
C22*A*—H22*A*···O11*B*^iv^	0.93	2.60	3.391 (5)	144
C22*B*—H22*B*···O11*A*^v^	0.93	2.56	3.390 (5)	149

Symmetry codes: (i) *x*−1/2, −*y*+1, *z*; (ii) *x*, *y*−1, *z*; (iii) *x*+1/2, −*y*, *z*; (iv) −*x*+1, −*y*+1, *z*+1/2; (v) −*x*+1, −*y*+1, *z*−1/2.
